# Comparison of the effectiveness of four Budyko-based methods in attributing long-term changes in actual evapotranspiration

**DOI:** 10.1038/s41598-018-31036-x

**Published:** 2018-08-23

**Authors:** Tingting Ning, Zhi Li, Qi Feng, Wenzhao Liu, Zongxing Li

**Affiliations:** 1 Key Laboratory of Ecohydrology of Inland River Basin, Northwest Institute of Eco-Environment and Resources, Chinese Academy of Sciences, Lanzhou, 730000 China; 2 College of Natural Resources and Environment, Northwest A&F University, Yangling, Shaanxi 712100 China; 3 State Key Laboratory of Soil Erosion and Dryland Farming on the Loess Plateau, Institute of Soil and Water Conservation, Chinese Academy of Sciences, Yangling, Shaanxi 712100 China

## Abstract

The responses of hydrological processes to climate change and anthropogenic influence have received significant attention over the past few decades. Several Budyko-based methods have been widely used to attribute hydrological variations and identify the extent of variation due to climate change and human activities. However, the accuracy of various methods has rarely been compared. This study employed four Budyko-based methods, namely the total differential method, complementary method, extrapolation method, and decomposition method, to attribute the changes in actual evapotranspiration in 13 basins in China’s Loess Plateau. We compared their performances and analysed factors that contribute to the differences in attribution results yielded by the various methods. The results showed that the total differential, complementary, and decomposition methods presented similar estimates of the contributions of climate change and human activities. However, the extrapolation method showed a large deviation in the contribution of human activities. The error of the extrapolation method was the largest, followed by that of the two-stage total differential method. The complementary method and decomposition method exhibited negligible errors.

## Introduction

As one of the major components of the hydrologic cycle, actual evapotranspiration (*ET*) drives energy and water exchanges between the hydrosphere, atmosphere and biosphere^1^. However, because of complexity of the land-plant-atmosphere system, direct measurement of *ET* is time consuming and expensive, and is hard to launch at regional scales^2,3^. Several methods have been proposed for evaluating regional *ET*, mainly including meteorology-driven diagnostic models, satellite data-driven approaches, energy-water balance methods, vegetation index-*ET* empirical relationship methods and statistical methods^4^. Among them, water balance method has been widely used because they are simple and sound in theory^5^. The long-term average water balance is in a steady state, and the water storage change in a catchment can be negligible. However, when it comes to shorter time scales, water storage change can be great^6^. To minimize the potential errors introduced by neglecting water storage variation, the hydrological year was introduced to estimate the annual *ET*^7–9^. In this way, the water input occurs mainly at the beginning of the year and the water is consumed within that year^9^.

Global hydrological research over the past decades has focussed on variations in the catchment water balance under changing environments where changes in processes such as runoff and *ET* have been assessed^10–12^. Climate change and human activities are considered as the major drivers that influence water resources and the catchment water cycle^13,14^. Quantification of the contributions of climate change and human activities on *ET* is important for efficient water resource management and ecosystem sustainability. Three kinds of approaches have been developed to evaluate the effects of climate change and human activities on hydrology, namely, the paired catchments approach, hydrological modelling, and statistical methods^12,15^. However, the accuracy of these methods has varied across applications^16–19^. For example, Jiang, *et al*.^20^ used three different methods namely, multi-regression, total differential method, and the hydrologic model to examine runoff variation in the Laohahe basin in north China^11^. They found that the contribution of human activities to runoff reduction estimated using the total differential method was slightly lower than values estimated by the other methods. Li, *et al*.^21^ applied two approaches including a sensitivity-based approach (comprising of a non-parametric model and six Budyko-based models) and hydrological modelling approach (employing the Xinanjiang and SIMHYD models) to three catchments in Australia. They concluded that the non-parametric method and Xinanjiang model underestimated the contribution of vegetation changes and overestimated the contribution of climate variability to the runoff reduction. Zhao, *et al*.^22^ attempted to quantify the impacts of human activities and climate change on runoff change in the middle reaches of the Yellow River. They noted that the results of the multi-regression method and total differential method presented different estimations for all the catchments assessed. Therefore, the issue of inconsistency in results derived using different methods has to be investigated. The accuracy of various methods in attributing runoff and *ET* variation should be compared and reasons and mechanisms contributing to the differences should be investigated as it can help us to understand the effects of climate change and human activities on the changes in hydrological processes more accurately.

The Budyko framework^23^ proposes that the long-term average water balance is controlled by the supply and demand of water from the atmosphere (represented by precipitation and potential evapotranspiration, respectively). The framework has recently been extensively applied in hydrological research due to its comprehensiveness and effectiveness in studying the effects of climate and surface condition on water resources^5,24,25^. Moreover, the framework does not depend on extensive historical climate and runoff data^26^. In past half-century, a number of Budyko equations have been proposed for the Budyko curve^5^. Among them, the Fu and Choudhury–Yang equations have been used widely. Recently, Zhou *et al*.^27^ found a generating function that can be used to produce any number of valid Budyko equations, which reflects the existence of functional relationship between these Budyko equations. Therefore, the similar performance of water balance variation estimation and its attribution could be obtained using different Budyko equations.

The Budyko-based attribution methods for catchment *ET*/runoff variation mainly include the total differential method^11^, extrapolation method^28^, decomposition method^29^, and complementary method^30^. However, most previous researches have only employed one of the Budyko-based methods to quantitatively identify the contributions of climate change and human activities on runoff/*ET* variation. Few researches have compared the similarities and differences of the attribution results estimated using different Budyko-based methods. Therefore, the objective of this study was to compare the effectiveness and applicability of these four different methods in estimating the relative contributions of climate change and human activities on *ET* variations in 13 basins in China’s Loess Plateau.

## Results

### Comparison of different total differential methods

The total differential method is one of the most popular attribution methods for water balance variation under the Budyko framework. *ET* change induced by a certain variable was indicated by multiplying the change value of the variable by its partial derivative (Eq.(6)). The partial derivatives can be estimated using the mean annual data for the entire study period^31–33^ or for the pre-change period^11,28^. In this study, we referred to the two methods as the whole-period method and forward-approximation method respectively. The average of the partial derivatives for the pre-change period and post-change period is also popularly used; we called it as the two-stage method. All three forms of the total differential method were employed in this study to identify the best-suited one.

**Figure 1:**
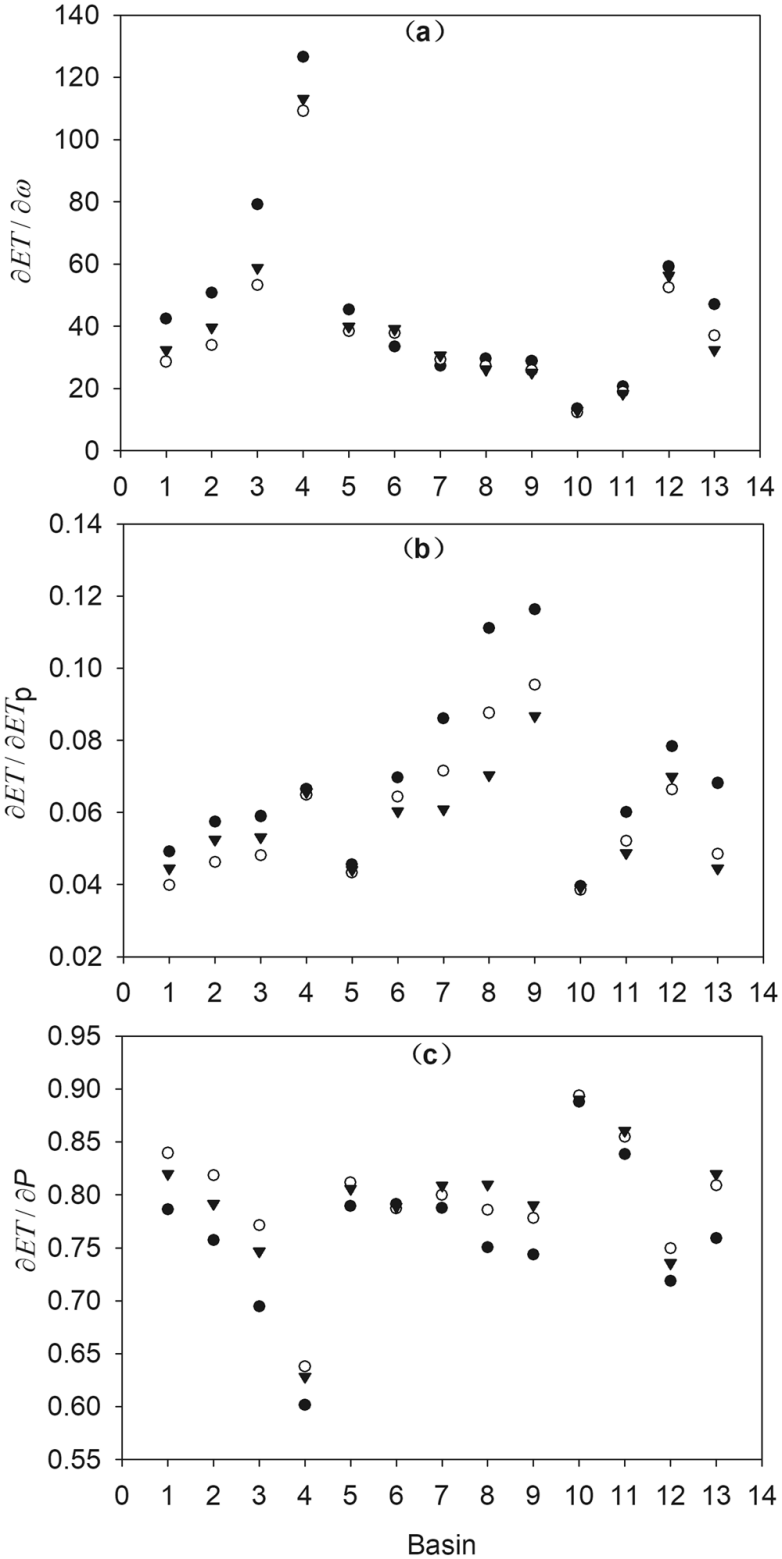
Comparison of partial derivative values of *ET* with (**a**) parameter *ω*, (**b**) *ET*_*p*_, and (**c**) *P* for the 13 basins using different total differentiation methods. The solid points, hollow points, and solid triangles represent the partial derivative values calculated using the forward-approximation method, two-stage method, and whole-period method, respectively. (Note: this figure was generated by Sigmaplot 10.0 (https://systatsoftware.com/)).

The partial derivative values of *ω*, *ET*_*p*_, and *P* estimated using the three total differential methods were compared graphically, as shown in Figure 1. As evident, the three groups of partial derivative values were different; specifically, the values for *ET*_*p*_ and *P* varied significantly between the methods. Moreover, the partial derivative values estimated using the forward-approximation method were compared with values calculated using the two-stage method; *∂ET/∂ET*_*p*_ was larger while *∂ET/∂P* was smaller using the forward-approximation method for all the catchments with an absolute mean difference of 17.8% and 4.3%, respectively. Further, *∂ET/∂ET*_*p*_ and *∂ET/∂P* estimated using the whole-period method was smaller than the values derived using the two-stage method for most catchments with an absolute mean difference of 8.5% and 1.6%, respectively.

The contributions of the three variables (*P*, *ET*_p_ and *ω*) to *ET* variation in the 13 basins were estimated using Eq. (6) (Table 1). The errors were evaluated by comparing the sum of contributions of the three variables (*C_sum*) and the actual change in *ET* (*∆ET*). Overall, the errors were the largest for the forward-approximation method, intermediate for the whole-period method, and smallest for the two-stage method at average values of 5.2 mm, 1.7 mm, and 0.5 mm across the 13 catchments, accounting for 16.4%, 5.7% and 1.4% of the observation respectively. The two-stage method appeared to be superior to the other methods in attributing *ET* variation. Thus, we selected it for comparison with the other three Budyko-based methods in section 3.2.

**Table 1:** Comparison of errors of the three total differential methods.

no	basin	Δ*ET*, mm	Forward-approximation			Two-stage			Whole-period		
			*C_sum*, mm	*ρ*1, mm	*ρ*2, %	*C_sum*, mm	*ρ*1, mm	*ρ*2, %	*C_sum*, mm	*ρ*1, mm	*ρ*2, %
1	Huangfu	−16.7	−12.3	4.4	−26.6	−16.4	0.3	−1.6	−19.1	−2.4	14.2
2	Gushan	29.5	37.7	8.2	27.7	30.1	0.6	2.2	33.1	3.6	12.3
3	Kuye	38.4	42.1	3.7	9.7	36.7	−1.7	−4.3	40.4	2.0	5.2
4	Tuwei	32.9	47.3	14.4	43.8	34.3	1.4	4.2	33.1	0.2	0.6
5	Wuding	−34.6	−31.4	3.2	−9.3	−34.6	0.0	−0.1	−36.5	−1.9	5.6
6	Qingjian	−43.6	−43.1	0.5	−1.2	−43.6	0.0	0.0	−43.7	−0.1	0.1
7	Yan	−46.1	−45.4	0.7	−1.4	−46.1	0.0	−0.1	−46.1	0.0	0.0
8	Beiluo	−52.6	−50.9	1.7	−3.2	−52.5	0.1	−0.1	−52.4	0.2	−0.3
9	Jing	−27.0	−24.4	2.6	−9.5	−27.0	0.0	−0.1	−26.5	0.5	−2.0
10	Fen	−34.7	−24.8	9.9	−28.5	−33.7	1.0	−3.0	−31.3	3.4	−9.8
11	Xinshui	−33.1	−25.6	7.5	−22.7	−32.6	0.5	−1.6	−32.1	1.0	−3.0
12	Sanchuan	−59.6	−57.2	2.4	−4.0	−59.6	0.0	0.0	−61.0	−1.4	2.3
13	Qiushui	−30.6	−22.8	7.8	−25.6	−30.3	0.3	−1.0	−36.2	−5.6	18.4

### Comparing the impacts of climate change

The estimated contributions of climate change on the variation in *ET* (Δ*ET*_*c*_) in the 13 basins calculated using the four Budyko-based attribution methods were compared. The results are listed in Table 2. Generally, the Δ*ET*_*c*_ estimated using the four methods were close. Moreover, the results of the total differential and complementary methods were the same, suggesting that all four methods were appropriate to calculate the contributions of climate change on *ET* variation. However, differences existed between these methods. The absolute mean relative difference between Δ*ET*_*c*_ calculated using the decomposition method and total differential method was 2.8%. Further, their values were higher than 5% in basins 1, 10, and 13. Δ*ET*_*c*_ calculated by the extrapolation method was smaller than the value calculated by the total differential method and complementary method in most of the basins with a mean relative difference of 5.4%; the value was up to 13% in basin 13.

**Table 2:** Contributions of climate change on *ET* variation assessed using four Budyko-based methods.

	Total differential method		Complementary method		Decomposition method		Extrapolation method	
	Δ*ET*_*c*_, mm	φ_c_, %	Δ*ET*_*c*_, mm	φ_c_,%	Δ*ET*_*c*_, mm	φ_c_,%	Δ*ET*_*c*_, mm	φ_c_,%
1	−30.1	180.2	−30.1	180.2	−29.3	175.2	−29.1	174.1
2	−10.3	−35.1	−10.3	−35.1	−9.3	−31.4	−9.2	−31.2
3	−9.5	−24.7	−9.5	−24.7	−8.1	−21.0	−7.9	−20.7
4	−9.8	−29.9	−9.8	−29.9	−9.0	−27.3	−9.0	−27.3
5	−40.2	116.2	−40.2	116.2	−39.6	114.4	−38.1	110.0
6	−36.7	84.2	−36.7	84.2	−37.4	85.8	−36.1	82.9
7	−41.5	90.0	−41.5	90.0	−42.0	91.2	−40.7	88.3
8	−51.8	98.5	−51.8	98.5	−51.9	98.7	−50.1	95.3
9	−31.6	116.9	−31.6	116.9	−31.2	115.4	−29.8	110.3
10	−46.5	133.9	−46.5	133.9	−44.8	129.2	−43.5	125.3
11	−44.6	134.9	−44.6	134.9	−43.3	130.7	−41.8	126.3
12	−55.0	92.3	−55.0	92.3	−55.6	93.3	−52.2	87.6
13	−43.8	143.1	−43.8	143.1	−42.3	138.2	−39.8	129.9

Note: φ_c_ is the relative contributions of climate change to ET variation, ${{\rm{\phi }}}_{{\rm{c}}}=({\rm{\Delta }}E{T}_{c}/C\_sum)\times 100 \%$.

During the assessment of the contribution of climate change on catchment water balance change, researchers attempt to identify whether the water condition (represented by *P*) or heat condition (represented by *ET*_*p*_) dominated the water balance change^34–36^. However, in this case, the contributions of *P* and *ET*_*p*_ cannot be separated further using the decomposition method and extrapolation method.

### Comparing the impacts of human activities

Similarly, *∆ET*_*h*_ values were also obtained by the total differential, complementary, and decomposition methods, and gaps were noted (Table 3). Unlike the values of *∆ET*_*c*_, *∆ET*_*h*_ values calculated by the total differential method were smaller than values calculated by the complementary method in most basins, with an absolute mean relative difference of 1.4%. The largest gap appeared in basins 5 and 6 with a difference of −4.4% and 4.1%. However, *∆ET*_*h*_ values calculated by the extrapolation method exhibited large difference compared to values calculated by the other three methods. For example, the absolute mean relative difference of ∆*ET*_*h*_ between the extrapolation method and complementary method was 31.7% and the relative difference in basins 8 and 15 were as high as 90.2% and 116.9%, respectively, suggesting that the extrapolation method was not suitable for estimating the contributions of human activities on *ET* variation in this study.

**Table 3:** Contribution of human activities on *ET* variation assessed using four Budyko-based methods.

	Total differential method		Complementary method		Decomposition method		Extrapolation method	
	Δ*ET*_*h*_, mm	φ_h_, %	Δ*ET*_*h*_, mm	φ_h_, %	Δ*ET*_*h*_, mm	φ_h_, %	Δ*ET*_*h*_, mm	φ_h_, %
1	13.7	−81.8	13.4	−80.2	12.6	−75.2	5.9	−35.4
2	40.5	137.2	39.8	135.1	38.8	131.4	38.6	130.9
3	46.2	120.4	47.9	124.8	46.5	121.0	48.4	126.1
4	44.1	134.2	42.8	130.1	41.9	127.5	45.9	139.6
5	5.6	−16.3	5.6	−16.1	5.0	−14.4	14.8	−42.8
6	−6.9	15.8	−6.9	15.9	−6.2	14.3	32.4	−74.3
7	−4.6	9.9	−4.6	10.0	−4.1	8.8	3.1	−6.6
8	−0.7	1.4	−0.8	1.5	−0.6	1.2	8.0	−15.3
9	4.6	−17.0	4.5	−16.8	4.1	−15.3	8.7	−32.2
10	12.8	−36.9	11.8	−33.9	10.1	−29.2	10.1	−29.0
11	12.1	−36.5	11.6	−34.9	10.2	−30.7	11.2	−33.8
12	−4.6	7.7	−4.6	7.8	−4.0	6.7	32.8	−55.1
13	13.5	−44.0	13.2	−43.1	11.7	−38.2	49.0	−160.0

Note: φ_h_ is the relative contributions of human activities to *ET* variation, ${\phi }_{{\rm{h}}}{=}{(}{\rm{\Delta }}{E}{{T}}_{{h}}{/}{C}{\_}{s}{u}{m}{)}\times {100}{ \% }$.

### Comparing errors of the four methods

The changes in *ET* calculated by the four methods, i.e. the sum of Δ*ET*_*c*_ and Δ*ET*_*h*_ were compared to the observed *ET* (*∆ET*). The results showed that the errors of the extrapolation method were the largest with mean relative value of 13.3%; the error exceeded 40% in basins 7, 13, and 14. The two-stage total differential method ranked next, with a mean relative value of 1.4%. The complementary and decomposition methods exhibited negligible errors. However, it should be noted that the decomposition method directly used the difference between Δ*ET* and Δ*ET*_*h*_ as Δ*ET*_*c*_, and its error was hidden in Δ*ET*_*c*_.

## Discussion

In this study, we compared the effectiveness of four Budyko-based methods in estimating the contributions of climate change and human activities on the variation in *ET*. The results highlighted that the estimated changes in the *ET* varied between methods because of their assumptions and structure. This section discusses some possible reasons for the difference in performance of each method.

### Uncertainties of the decomposition and total differential methods

The decomposition method hinges on two assumptions. Firstly, *ET*/*P* follows the same Budyko curve as the pre-change period, which implies that the controlling parameters in the Budyko equations are constant. However, Ning, *et al*.^9^ reported that the values of the parameter *ω* in all the basins have exhibited an upward trend, and this trend has been more dramatic after the 1980 s. For example, in the Huangfu basin (no. 1), the mean annual *ω* in the pre-change and post-change periods was 2.32 and 2.56, respectively, and their difference was 0.24. The mean partial derivative coefficient of *ET* to *ω* was 46.9. Thus, if the *ω* values of different periods were used to calculate the contribution of human activities to ET variation, the error would increase to 11.3 mm. Therefore, the constant controlling parameter is an error source, and also applies to the total differential method and complementary method. Secondly, the decomposition method also assumed that *P* and *ET*_*P*_ are stationary during the pre-change and post-change periods^29^. However, both natural climate variability and human activities can induce change in *P* and *ET*_*P*_ at the interdecadal or interannual scales.

Moreover, the partial derivative coefficients in the total differential method should be calculated using the complete Taylor expansion to deliver accurate results. However, in our study, the partial derivative coefficients were mostly estimated as a first-order approximation. Yang, *et al*.^37^ demonstrated that ignoring the higher orders of the Taylor expansion will underestimate the contribution of climate change to hydrological processes when precipitation increases or potential *ET* decreases. Furthermore, the forward-approximation and whole-period total differential methods actually assume that the partial derivative coefficients of *ET* to *P*, *ET*_*p*_, and *ω* did not change during the study period. However, the annual partial derivative coefficients for the period 1961–2010 for the 13 basins calculated in this study showed that the partial derivative coefficients of *ET* to *ET*_*p*_ and *ω* in all the basins exhibited significant downward trend from 1960 to 2010 (p < 0.05). Conversely, the partial derivative coefficients of *ET* to *P* in all the basins showed significant upward trend (p < 0.05). This implies that using the constant partial derivative coefficients could result in large errors in the estimated *∆ET*.

### Possible reason for large error of the extrapolation method

A comparison of the error in Δ*ET*_*h*_ derived using the four methods indicated that the largest derivation was produced by the extrapolation method. The extrapolation method used the sensitivity analysis and the trend analysis to evaluate the effects of climate effect and human activities, respectively. And the large error of this method was mainly induced by the trend analysis. Specifically, early occurrence of the abrupt year resulted in small sample size for the relationship development in Eq. (9), and subsequently led to errors when the relationship was applied to the post-change period. Thus, the unstable relationship between *ET* and *P* in the pre-change period is the most dominating factor leads to the inaccurate result in this study. To address the problem of small sample size in the pre-change period, Zhang, *et al*.^28^ used monthly runoff and precipitation to build the relationship. In most cases, annual *ET* was obtained by ignoring water storage change, which was acceptable if this was carried out for multiple hydrological years^7–9^. If the relationship between *ET* and *P* is built on a monthly scale, *ET* estimation would exhibit higher uncertainty as water storage variations on a monthly scale cannot be ignored. This argument indicates that even when the sample size is large enough and the determining coefficient of the developed relationship is high, the errors would be large. Further, climate change may make some contribution to Δ*ET*_*h*_ in this method and induce the attribution error. For instance, the *P*_*post*_ series was used to predict the *ET*_*post*_; however, the *P*_*post*_ itself must have already been influenced by the climate change.

### Other potential factors influencing attribution results

Many previous studies^25,31,32^ have considered that the controlling parameters of the Budyko model represent the effects of the land use/cover change (i.e. human activities) completely. However, several researches have highlighted that climate seasonality also strongly affects the controlling parameters of the Budyko models^9,30,37,38^. In particular, our previous study found that parameter *ω* was well-correlated with vegetation coverage and climate seasonality, and if the impacts of climate seasonality on *ET* were ignored, the contribution of vegetation (i.e. land use/cover) will be estimated with a large error^9^. Thus, the effects of climate seasonality should be considered when attribution analysis of catchment runoff or *ET* variation is conducted. Further, although the attribution equation requires the variables to be independent, major factors such as potential evapotranspiration, precipitation, and controlling parameters can interact with each other, increasing uncertainties.

## Conclusions

The water cycle in many regions of the world has been affected by climate change and human activities and has experienced drastic changes in the recent decades. Budyko-based methods are popularly used to detect hydrological variation attributed to climate change and human activities due to their simplicity and clear physical foundations. The methods mainly include the total differential, complementary, extrapolation, and decomposition methods. However, little attention has been paid to assessing and comparing the effectiveness of these methods. The study obtained the annual evapotranspiration data of 13 typical basins in China’s Loess Plateau from 1961 to 2012 using the hydrological year approach, and compared the performance of the various Budyko-based methods in quantifying the relative contributions of related environmental/anthropogenic factors to long-term *ET* variation. The following main conclusions were drawn: in the total differential method, use of average values of the partial derivatives for the pre-change and post-change periods to conduct attribution analysis of *ET* variation is better than using long-term mean annual data for the whole study period or for the pre-change period. The contribution of climate change (*∆ET*_*c*_) and human activities (*∆ET*_*h*_) estimated by the total differential, complementary, and decomposition methods were similar. Similar *∆ET*_*c*_ could also be obtained by the extrapolation method. However, *∆ET*_*h*_ values calculated by this method exhibited large difference compared to the other three methods. The error of the extrapolation method was largest, followed by the two-stage total differential method. The complementary and decomposition methods recorded negligible errors.

## Data and Methods

### Study area

**Figure 2:**
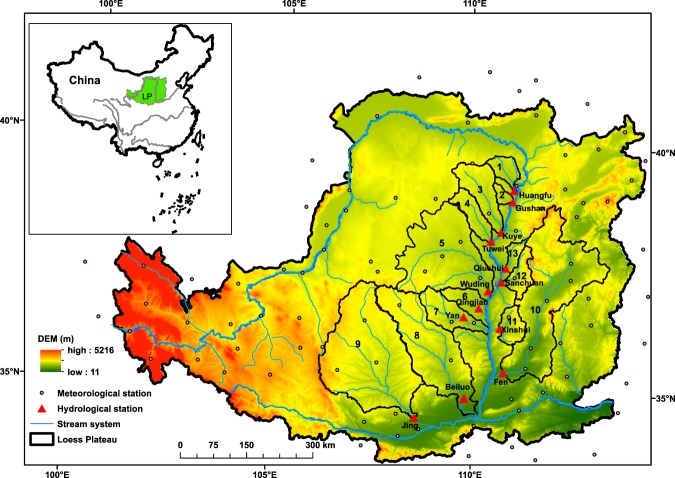
Map highlighting the 13 catchments in China’s Loess Plateau assessed in this study. (Note: the map was generated with licensed ArcGIS 10.1 (http://www.esri.com) using the data from the Geospatial Data Cloud site, Computer Network Information Center, Chinese Academy of Sciences (http://www.gscloud.cn/)).

The Loess Plateau is located in the upper and middle reaches of the Yellow River in North China (Figure 2), and extends over an area of 6.4 × 10^5^ km^2^. Most areas experience semi-arid and sub-humid climate. Based on the daily meteorological data of 96 stations in the Loess Plateau from 1961 to 2012, the spatial and temporal change trend of precipitation (*P*), mean temperature (*T*) and potential evapotranspiration for whole Loess Plateau were exhibited. Spatially, *P* decreases along the southeast-northwest direction, ranging from 200 mm to 750 mm, 80% of which is distributed during June to September. *T* decreases from the southeast to northwest, ranging from 1.5–15 °C. *ET*_*P*_, calculated by the method of Priestley and Taylor^39^, distinctly decreases in three directions, namely, northwest-east, northwest-south, and northwest-southwest, ranging from 812–1235 mm. However, temporal variation in these variables has been noted; P is decreasing slowly by 5.2 mm/decade (p > 0.05); while T has increased significantly by 0.3 °C/decade (p < 0.05). *ET*_*P*_ has exhibited a downward trend at −3.1 mm/decade (p > 0.05). The soil erosion in the study area is very heavy as it is influenced by scarce and unevenly distributed rainfall, steep landscape, highly erodible loessial soil, low canopy cover degree, and long history of intensive cultivation. Human activities in this region are also intensive, mainly refers to the implementation of soil and water conservation measures. The construction of energy bases and new towns have also significantly altered the land surface and consequently affected the regional water cycle.

### Data

Monthly runoff records for the 13 catchments from 1961 to 2012 were obtained from the Yellow River Conservancy Commission. Detailed characteristics of each catchment are presented in Table S1 in the Supporting information. Daily meteorological data for 96 stations, including precipitation, temperature, sunshine hours, and relative humidity for the period 1961–2012, was acquired from the China Meteorological Administration.

## Methods

### Abrupt point test

The non-parametric Pettitt method was used to detect the abrupt year of *ET*; detailed procedure can be found in Mallakpour and Villarini^40^. Once an abrupt year is identified, the entire study period is divided into two sub-periods: 1961 to the abrupt year, referring to as the pre-change period, represented the initial state of *ET* when no significant human activities occurred; and the residual data period, referring to as the post-change period, represented changed *ET* and is associated with significant human activities.

### Attributing changes in *ET*

The catchment water balance can be expressed as:

1 $$ET=P-R-{\rm{\Delta }}S$$

where *R* and Δ*S* are the runoff and change in water storage respectively. Δ*S* can be ignored for long-term assessments but can be significant for assessments conducted for shorter time scales. Thus, the hydrological year concept (July to June of the following year)^9^ was introduced to assess the annual *P* and *R* to minimize the errors that may occur in the annual *ET* calculation due to the exclusion of Δ*S*

To estimate *ET*, the Budyko formula expressed by the Fu equation was used^41^:

2 $$\begin{array}{c}\frac{ET}{P}=1+\frac{E{T}_{p}}{P}-{[1+{(\frac{E{T}_{p}}{P})}^{\omega }]}^{1/\omega }\,or\\ \frac{ET}{E{T}_{p}}=1+\frac{P}{E{T}_{p}}-{[1+{(\frac{P}{E{T}_{p}})}^{\omega }]}^{1/\omega }\end{array}$$

where *ω* is the controlling parameter that determines the shape of the Budyko curve, which is used to represent the effects of land use/cover change induced by human activities in the long-term. *ET*_*P*_ is the potential evapotranspiration calculated using the equation suggested by Priestley and Taylor^39^.

The change in *ET* from pre-change period to post-change period can be described as:

3 $${\rm{\Delta }}ET=E{T}_{post}-E{T}_{pre}$$

where *ET*_*post*_ and *ET*_*pre*_ are the mean annual *ET* values during the post-change and pre-change periods respectively.

The total contributions of climate change and human activities can be expressed as:

4 $$C\_sum={\rm{\Delta }}E{T}_{c}+{\rm{\Delta }}E{T}_{h}$$

where Δ*ET*_*c*_ and Δ*ET*_*h*_ are climate-induced and human-induced ET changes, respectively.

The absolute and relative errors between *C*_*sum* and Δ*ET* are calculated as:

5a $${\rho }_{1}={C}_{sum}-{\rm{\Delta }}ET$$

5b $${\rho }_{2}=\frac{{C}_{sum}-{\rm{\Delta }}ET\,}{{\rm{\Delta }}ET}\times 100 \% $$

### Budyko-based attribution methods

Four methods, namely, the total differential method, complementary method, extrapolation method, and decomposition method were used to calculate Δ*ET*_*c*_ and Δ*ET*_*h*_.

1. Total differential method
  Assuming that *ET*_*P*_, *P*, and parameter *ω* in Eq. (1) were independent variables, the total differential of *ET* can be used to evaluate the contributions of major climate factors and human activities on the long-term scale^2^:
  6a $$dET=\frac{\partial ET}{\partial P}dP+\frac{\partial ET}{\partial E{T}_{P}}dE{T}_{P}+\frac{\partial ET}{\partial \omega }d\omega $$
  Ignoring the higher orders of the Taylor expansion in Eq. (6a), the equation can be rewritten as:
  6b $${\rm{\Delta }}ET\approx \frac{\partial ET}{\partial P}{\rm{\Delta }}P+\frac{\partial ET}{\partial E{T}_{p}}{\rm{\Delta }}E{T}_{p}+\frac{\partial ET}{\partial \omega }{\rm{\Delta }}\omega $$
  where Δ*P*, Δ*ET*_*p*_, and Δ*ω* are the changes in the annual *P*, *ET*_*p*,_ and *ω* from the pre-change period to post-change period, respectively. The values of $(\frac{\partial ET}{\partial P}{\rm{\Delta }}P+\frac{\partial ET}{\partial E{T}_{p}}{\rm{\Delta }}E{T}_{p})$ and $\frac{\partial ET}{\partial \omega }{\rm{\Delta }}\omega$ represent the contributions of climate change and human activities on *ET* variation, i.e. ${\rm{\Delta }}E{T}_{c}\,{\rm{and}}\,{\rm{\Delta }}E{T}_{h}$ in Eq. (4), respectively. The three partial derivatives can be calculated as:
  7a $$\frac{\partial ET}{\partial P}=1-{[1+{(\frac{P}{E{T}_{p}})}^{\omega }]}^{\frac{1}{\omega }-1}{(\frac{P}{E{T}_{p}})}^{\omega -1}$$
  7b $$\frac{\partial ET}{\partial E{T}_{p}}=1-{[{(\frac{E{T}_{p}}{P})}^{\omega }]}^{\frac{1}{\omega }-1}{(\frac{E{T}_{p}}{P})}^{\omega -1}$$
  7c $$\frac{\partial ET}{\partial \omega }=-\,{(E{T}_{p}^{\omega }+{P}^{\omega })}^{\frac{1}{\omega }}\times [-\frac{\mathrm{ln}(E{T}_{p}^{\omega }+{P}^{\omega })}{{\omega }^{2}}+\frac{1}{\omega }\frac{E{T}_{p}^{\omega }\,\mathrm{ln}(E{T}_{p})+{P}^{\omega }\,\mathrm{ln}(P)}{E{T}_{p}^{\omega }+{P}^{\omega }}]$$
2. Complementary method
  The complementary method was introduced by Zhou, *et al*.^30^ to reduce the errors of the total differential method caused by the exclusion of the higher orders of Taylor expansion^21^. It can be expressed as:
  8a $$\begin{array}{rcl}{\rm{\Delta }}ET & = & \alpha [{(\frac{\partial ET}{\partial P})}_{pre}{\rm{\Delta }}P+{(\frac{\partial ET}{\partial E{T}_{p}})}_{pre}{\rm{\Delta }}E{T}_{p}+{P}_{post}{\rm{\Delta }}(\frac{\partial ET}{\partial P})+E{T}_{p,post}{\rm{\Delta }}(\frac{\partial ET}{\partial E{T}_{p}})]\\  &  & +\,(1-\alpha )[{(\frac{\partial ET}{\partial P})}_{post}{\rm{\Delta }}P+{(\frac{\partial ET}{\partial E{T}_{p}})}_{post}{\rm{\Delta }}E{T}_{p}+{P}_{pre}{\rm{\Delta }}(\frac{\partial ET}{\partial P})+E{T}_{p,pre}{\rm{\Delta }}(\frac{\partial ET}{\partial E{T}_{p}})]\end{array}$$
  where *α* is a weighting coefficient that varies from 0 to 1, which can determine the bounds of *P*, *ET*_*p*,_ and *ω* effect. In this study, we defined *α* = 0.5 according to the recommendation of Zhou, *et al*.^30^. The subscripts pre and post represent the value of the variable during the pre-change and post-change periods, respectively. ${\rm{\Delta }}(\frac{\partial ET}{\partial P})$ and ${\rm{\Delta }}(\frac{\partial ET}{\partial E{T}_{p}})$ are the changes in the $\frac{\partial ET}{\partial P}$ and $\frac{\partial ET}{\partial E{T}_{P}}$ from the pre-change period to post-change period, respectively. Based on Eq. (8a), the contributions of climate change and human activities can be expressed as:
  8b $${\rm{\Delta }}E{T}_{c}=\alpha [{(\frac{\partial ET}{\partial P})}_{pre}{\rm{\Delta }}P+{(\frac{\partial ET}{\partial E{T}_{p}})}_{pre}{\rm{\Delta }}E{T}_{p}]+(1-\alpha )[{(\frac{\partial ET}{\partial P})}_{post}{\rm{\Delta }}P+{(\frac{\partial ET}{\partial E{T}_{p}})}_{post}{\rm{\Delta }}E{T}_{p}]$$
  8c $${\rm{\Delta }}E{T}_{h}=\alpha [{P}_{post}{\rm{\Delta }}(\frac{\partial ET}{\partial P})+E{T}_{p,post}{\rm{\Delta }}(\frac{\partial ET}{\partial E{T}_{p}})]+(1-\alpha )[{P}_{pre}{\rm{\Delta }}(\frac{\partial ET}{\partial P})+E{T}_{p,pre}{\rm{\Delta }}(\frac{\partial ET}{\partial E{T}_{p}})]$$
3. Extrapolation method
  The relationship between *P* and *ET* during the pre-change period is considered as:
  9 $$E{T}_{pre}=f({P}_{pre})$$
  Specifically, the function *f* for each catchment was fitted by the annual *ET* and *P* series using the linear regression method.
  Next, the function *f* was applied to the post-change period as:
  10 $$E{T}_{post}^{^{\prime} }=f({P}_{post})$$
  Thus, the contribution of human activities on *ET* can be written as:
  11 $${\rm{\Delta }}E{T}_{h}=\overline{E{T}_{post}}-\overline{E{T}_{post}^{^{\prime} }}$$
  where $\overline{E{T}_{post}}$ and $\overline{E{T}_{post}^{^{\prime} }}$ are the mean annual values of actual and predicted *ET* during the post-change period respectively.
  Further, Δ*ET*_*c*_ was calculated using the forward approximation method.
4. Decomposition method

The decomposition method is presented in Fig. S1 in the Supporting information. Ignoring the intra-annual variability of *P*, the contribution of human activities to runoff change was defined as:

12 $${\rm{\Delta }}{Q}_{h}={P}_{post}(\frac{E{T}_{post}^{^{\prime} }}{{P}_{post}}-\frac{E{T}_{post}}{{P}_{post}})$$

Similarly, the contribution of human activities to variation in *ET* can be expressed as:

13 $${\rm{\Delta }}E{T}_{h}=E{T}_{post}-E{T}_{post}^{^{\prime} }$$

where $E{T}_{post}^{^{\prime} }$ is the mean annual ET calculated by Eq. (2) using the mean annual *P* and *ET*_*p*_ during the post-change period and parameter *ω* during pre-change period.

Δ*ET*_*c*_ can be calculated as:

14 $${\rm{\Delta }}E{T}_{c}={\rm{\Delta }}ET-{\rm{\Delta }}E{T}_{h}$$

## Electronic supplementary material

Supplementary information
