# Supplementary material for: Comparison of the effectiveness of four Budyko-based methods in attributing long-term changes in actual evapotranspiration
Source: Sci Rep. 2018 Aug 23;8:12665. doi: 10.1038/s41598-018-31036-x (PMC6107678; doi:10.1038/s41598-018-31036-x)
Supplement: Supplementary file 1 — Supplementary information [file 41598_2018_31036_MOESM1_ESM.pdf]

# Comparison of the effectiveness of four Budyko-based methods in attributing long-term changes in actual evapotranspiration

Tingting Ning[1,3], Zhi Li[2], Qi Feng[1*], Wenzhao Liu[3*], and Zongxing Li[1]

[1]*Key Laboratory of Ecohydrology of Inland River Basin, Northwest Institute of Eco-Environment and Resources, Chinese Academy of Sciences, Lanzhou 730000, China*

[2]*College of Natural Resources and Environment, Northwest A&F University, Yangling, Shaanxi 712100, China*

[3]*State Key Laboratory of Soil Erosion and Dryland Farming on the Loess Plateau, Institute of Soil and Water Conservation, Chinese Academy of Sciences, Yangling, Shaanxi 712100, China*

Table S1 Hydro-meteorological characteristics of the catchments during 1961–2012

| | | | Pre-change period | | | | Post-change period | | | |
|---|---|---|---|---|---|---|---|---|---|---|
| No. | Basin name | abrupt year | ET (mm) | P (mm) | $ET_p$ (mm) | ω | ET (mm) | P (mm) | $ET_p$ (mm) | ω |
| 1 | Huangfu | 1970(**) | 364.1 | 417.5 | 1025.1 | 2.32 | 347.4 | 380.4 | 985.0 | 2.56 |
| 2 | Gushan | 2001(ns) | 349.3 | 408.4 | 997.4 | 2.23 | 378.8 | 395.4 | 994.3 | 3.17 |
| 3 | Kuye | 2001(ns) | 318.6 | 389.3 | 1029.5 | 2.00 | 354.0 | 377.0 | 1039.6 | 2.88 |
| 4 | Tuwei | 1985(**) | 282.0 | 401.9 | 1050.2 | 1.68 | 314.9 | 387.1 | 1031.8 | 1.98 |
| 5 | Wuding | 1970(**) | 391.6 | 443.3 | 1030.0 | 2.45 | 355.7 | 387.2 | 1049.3 | 2.55 |
| 6 | Qingjian | 1986(**) | 455.0 | 495.7 | 998.5 | 2.96 | 411.4 | 447.0 | 1014.0 | 2.80 |
| 7 | Yan | 1986(**) | 469.6 | 506.6 | 981.0 | 3.16 | 423.5 | 453.5 | 989.5 | 3.03 |
| 8 | Beiluo | 1986(**) | 518.3 | 554.7 | 966.2 | 3.54 | 462.8 | 486.3 | 969.4 | 3.52 |
| 9 | Jing | 1991(**) | 517.8 | 561.4 | 927.8 | 3.48 | 490.7 | 518.6 | 947.0 | 3.64 |
| 10 | Fen | 1991(**) | 479.8 | 510.0 | 986.2 | 3.39 | 442.2 | 452.3 | 996.1 | 4.05 |
| 11 | Xinshui | 1986(**) | 489.9 | 530.2 | 991.4 | 3.19 | 456.9 | 474.9 | 999.2 | 3.63 |
| 12 | Sanchuan | 1970(**) | 458.3 | 534.4 | 985.1 | 2.56 | 402.7 | 456.0 | 1001.1 | 2.49 |
| 13 | Qiushui | 1965(**) | 452.3 | 520.2 | 982.4 | 2.61 | 421.8 | 457.1 | 1011.6 | 2.86 |

note: ** indicates abrupt point was significant at the level of p = 0.01; ns means the abrupt point was not significant (p>0.05).

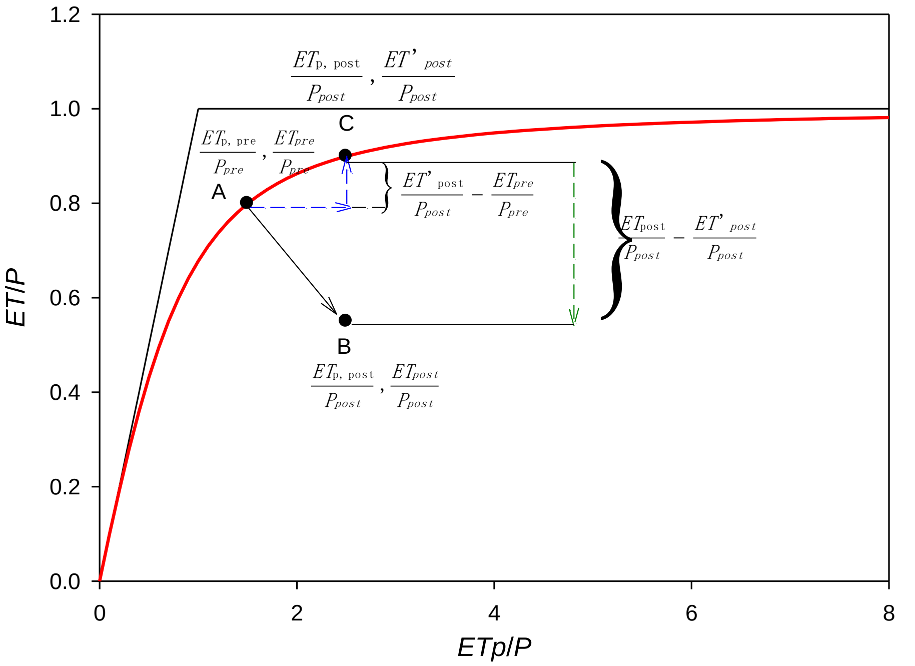


Fig. S1 Decomposition method used to distinguish between human and climate contributions to the mean annual runoff change. A catchment shifts from point A (pre-period) to B (post-period) affected by both human activities and climate change; while under climate change only, it evolves from A to C. Thus, human activities cause a vertical shift ($ET'_{post}/P_{post}$ to $ET_{post}/P_{post}$); climate change causes both vertical ($ET_{pre}/P_{pre}$ to $ET'_{post}/P_{post}$) and horizontal ($ET_{p,pre}/P_{pre}$ to $ET_{p,post}/P_{post}$) shifts.
